# Kinetics on Demand Is a Simple Mathematical Solution that Fits Recorded Caffeine-Induced Luminal SR Ca^2+^ Changes in Smooth Muscle Cells

**DOI:** 10.1371/journal.pone.0138195

**Published:** 2015-09-21

**Authors:** Norma C. Perez-Rosas, Norma L. Gomez-Viquez, Adan Dagnino-Acosta, Moises Santillan, Agustín Guerrero-Hernandez

**Affiliations:** 1 Unidad Monterrey, Cinvestav, Apodaca, Nuevo Leon, Mexico; 2 Departamento de Farmacobiología, Unidad Sur, Cinvestav, Mexico City, Mexico; 3 Centro Universitario de Investigaciones Biomédicas, Universidad de Colima, Colima, Colima, Mexico; 4 Departamento de Bioquímica, Cinvestav, Mexico City, Mexico; University of Newcastle, AUSTRALIA

## Abstract

The process of Ca^2+^ release from sarcoplasmic reticulum (SR) comprises 4 phases in smooth muscle cells. Phase 1 is characterized by a large increase of the intracellular Ca^2+^ concentration ([Ca^2+^]_i_) with a minimal reduction of the free luminal SR [Ca^2+^] ([Ca^2+^]_FSR_). Importantly, active SR Ca^2+^ ATPases (SERCA pumps) are necessary for phase 1 to occur. This situation cannot be explained by the standard kinetics that involves a fixed amount of luminal Ca^2+^ binding sites. A new mathematical model was developed that assumes an increasing SR Ca^2+^ buffering capacity in response to an increase of the luminal SR [Ca^2+^] that is called Kinetics-on-Demand (KonD) model. This approach can explain both phase 1 and the refractory period associated with a recovered [Ca^2+^]_FSR_. Additionally, our data suggest that active SERCA pumps are a requisite for KonD to be functional; otherwise luminal SR Ca^2+^ binding proteins switch to standard kinetics. The importance of KonD Ca^2+^ binding properties is twofold: a more efficient Ca^2+^ release process and that [Ca^2+^]_FSR_ and Ca^2+^-bound to SR proteins ([Ca^2+^]_BSR_) can be regulated separately allowing for Ca^2+^ release to occur (provided by Ca^2+^-bound to luminal Ca^2+^ binding proteins) without an initial reduction of the [Ca^2+^]_FSR_.

## Introduction

The main internal Ca^2+^ store is the sarcoplasmic reticulum (SR) in muscle cells and the endoplasmic reticulum (ER) in non-muscle cells. These membrane organelles are distributed throughout the cell and are endowed with three main elements 1) SERCA pumps, 2) different types of Ca^2+^ release channels [Ryanodine or inositol 1,4,5-trisphosphate receptors (RyRs and IP_3_Rs, respectively)], and 3) luminal Ca^2+^-binding proteins (calsequestrin or calreticulin). All these different types of proteins produce and modulate Ca^2+^ release events that can be either localized in nature or traveling Ca^2+^ waves [1].

SERCA pumps refill internal Ca^2+^ stores via ATP hydrolysis [2]. These ATPases are regulated by the free luminal SR/ER [Ca^2+^] ([Ca^2+^]_FSR_) and by different type of proteins [3]. Although the role of SERCA pumps in Ca^2+^ refilling of SR stores is well established, there are also data indicating that SERCA pumps facilitate Ca^2+^ release by a mechanism unrelated to the replenishment of internal Ca^2+^ stores [4]. Ikemoto´s group has shown that activation of RyRs produces an increase in the turnover rate of SERCA pumps before any reduction in the [Ca^2+^]_FSR_ [5]. Additionally, they have found that low concentrations of polylysine that activate a small number of RyRs, can induce an initial increase in the luminal SR Ca^2+^ level [6] and activation of SERCA pumps [5,7]. In pancreatic acinar cells, it was shown that inhibition of SERCA pumps with thapsigargin decreases the rate of Ca^2+^ release and eliminates [Ca^2+^]_i_ gradients before any reduction of the ER Ca^2+^ store [8]. More recently, it was reported that rapid inhibition of SERCA pumps by UV-mediated uncaging of thapsigargin produces a significantly slower Ca^2+^ wave in heart cells [9]. In HeLa cells, the rapid inhibition of SERCA pumps to avoid any reduction of the [Ca^2+^]_FSR_, reduces the histamine-induced potentiation of IP_3_-induced Ca^2+^ release [10]. In smooth muscle cells, thapsigargin decreases the amplitude and the rate of rise of caffeine- and carbachol-induced [Ca^2+^]_i_ responses [4,11]. Moreover, inhibition of SERCA pumps decreased the coordinated Ca^2+^ release process even in overloaded Ca^2+^ stores of smooth muscle cells [12]. Interestingly, there is also evidence that increased activity of SERCA pumps enhances Ca^2+^ release. For instance, RGS2^-/-^ knockout displayed facilitation in the agonist-induced Ca^2+^ release, but due to increased expression of SERCA pump instead of the expected, but absent, larger IP_3_ production [13]. Overexpression of β adrenoceptors increases both SERCA pump activity and Ca^2+^ spark frequency in heart cells, in the absence of an overloaded SR Ca^2+^ store [14]. Collectively, these data suggest that SERCA pumps play an important role not only in refilling internal Ca^2+^ stores but also, in facilitating activation of release channels.

SR and ER also contain a large variety of proteins that bind Ca^2+^ with low affinity and high capacity, for instance calsequestrin and calreticulin. Interestingly, the Ca^2+^ buffer capacity of calsequestrin increases with the degree of polymerization, from monomer to dimer and to polymer [15]. These characteristics of calsequestrin suggest a possible explanation for the observation that SR and ER are able to supply large quantities of Ca^2+^ with minimal reductions in the [Ca^2+^]_FSR_ [11,16–19] i.e. the transition from dimer to monomer, for instance, releases a large quantity of Ca^2+^ with minimal modification of the [Ca^2+^]_FSR_ [15]. The presence of these concealed sources of Ca^2+^ is evident in cardiac cells as well, because the overexpression of calsequestrin containing a point mutation (CASQ2^R33Q^) produces Ca^2+^ sparks of larger amplitude, although the SR seems to be partially depleted because a reduced resting [Ca^2+^]_FSR_ is observed [20]. Additionally, the reduction in the [Ca^2+^]_FSR_ is similar for the smaller Ca^2+^ sparks than for the larger Ca^2+^ waves [17]. All these data imply that the change in [Ca^2+^]_FSR_ cannot be correlated with the total amount of Ca^2+^ released by the SR so we have hypothesized that there is a concealed source of luminal Ca^2+^ for release channels [16].

Since it is not feasible yet to look simultaneously at both [Ca^2+^]_FSR_ and the amount of Ca^2+^-bound to luminal proteins ([Ca^2+^]_BSR_), to test this hypothesis a simple deterministic mathematical model was developed, in order to understand how release channels (particularly RyRs), luminal SR proteins and SERCA pumps work together to facilitate Ca^2+^ release in smooth muscle cells. This new model suggests that luminal SR Ca^2+^ binding proteins are working different to what it is commonly believed, since the number of apparent Ca^2+^ binding sites increases as a function of the free luminal SR [Ca^2+^], this situation we have called Kinetics on Demand (KonD), and requires active SERCA pumps to have an efficient Ca^2+^ release event.

## Materials and Methods

### Ethics statement on animal use

All animal care and experimental procedures were performed in compliance with the Mexican Official Norm for the Use and Care of Laboratory Animals (NOM-062-ZOO-1999) and the protocol was approved by the local Ethics Committee on Animal Experimentation (CICUAL-Cinvestav) with the reference number 0306–06 and renewed with the reference number 0131–15. Animals were bred and housed in Cinvestav Animal facility with clean air and controlled both light and temperature. Food and water was given ad libitum. Precautions were implemented to minimize animal use and reduce pain and distress.

### Cell isolation and [Ca^2+^]_i_ recordings

Male albino guinea pigs of 400 to 490g weight (all from Cinvestav animal facility) were killed by decapitation followed by exsanguination. Single smooth muscle cells were isolated from guinea pig urinary bladder and loaded with fura-2 to record [Ca^2+^]_i_ as previously reported [4]. Simultaneous recording of the [Ca^2+^]_i_ and the free luminal SR Ca^2+^ level ([Ca^2+^]_FSR_) in these cells was carried out using fura-2 and Mag-Fluo-4, respectively; as previously described [16]. Ca^2+^ release was induced by application of 2 or 20 mM caffeine with a puffer pipette [4]. Inhibition of SERCA pump with short pulses of thapsigargin was carried out as previously described [4]. The development of the mathematical model is described below.

### Model development

For the purpose of the present model we have considered that smooth muscle cells consist of two compartments: the cytoplasm and the SR (S1 Fig), and [Ca^2+^]_Ti_ and [Ca^2+^]_TSR_ represent the total [Ca^2+^] in the respective compartments. Let J_1_ be the Ca^2+^ flux from the cytoplasm to outside the cell together with all cytoplasmic Ca^2+^ removal mechanisms (except SERCA pumps) that are involved in keeping a constant basal concentration of this ion, J_2_ be the Ca^2+^ flux from the SR to the cytoplasm via RyRs present in the SR membrane, and J_3_ be the Ca^2+^ flux from the cytoplasm to the SR via SERCA pumps located in the SR membrane. It follows from these considerations that

$$\frac{d{\left[Ca^{2+}\right]}_{Ti}}{dt}=-J_{1}+J_{2}-J_{3},$$ (1.1)

$$\frac{d{\left[Ca^{2+}\right]}_{TSR}}{dt}=\frac{J_{3}-J_{2}}{\gamma }.$$ (1.2)

All fluxes have units of concentration/time which are referred to the volume of the cytoplasm. Accordingly, the factor γ denotes the volume ratio between the SR and the cytoplasm.

As indicated above, J_1_ considers all plasma membrane Ca^2+^ removal mechanisms and based on the observation that this process has a first order kinetics, as shown by Guerrero et al. [21], we have assumed that

$$J_{1}=a\left[{\left[Ca^{2+}\right]}_{i}-\bar{{\left[Ca^{2+}\right]}_{i}}\right],$$ (1.3)

where [Ca^2+^]_i_ is the free cytoplasmic [Ca^2+^], while ${\bar{\left[Ca^{2+}\right]}}_{i}$ is the resting basal [Ca^2+^]_i_, and *a* is the first order kinetic constant for this flux. We have assumed that no membrane potential exists across the SR membrane [22], therefore the flux J_2_ is driven by the gradient of free Ca^2+^ ions across this membrane:

$$J_{2}=b\gamma^{n_{v}}P_{o}\left({\left[Ca^{2+}\right]}_{i},\left[Caff\right]\right)\left[{\left[Ca^{2+}\right]}_{FSR}-{\left[Ca^{2+}\right]}_{i}\right],$$ (1.4)

where *b* is a constant parameter proportional to the maximum [Ca^2+^] flow through a single RyR, *P*_*o*_([*Ca* ^2+^]_*i*_,[*Caff*]) is the probability that a single RyR is open as a function of [Ca^2+^]_i_ and of the caffeine concentration ([Caff]) used to induce Ca^2+^ release from SR store, [Ca^2+^]_FSR_ is the concentration of free Ca^2+^ in the SR, and $\gamma^{n_{v}}$ is a proportionality constant for the number of RyR based on *γ* being the volume ratio between SR and cytoplasm. SR appears to have a complex fractal-like structure, hence its surface should scale with respect to the volume according to a power law $\gamma^{n_{v}}$, where *n*_*v*_ is a parameter related to the SR fractal dimension [23]. Accordingly, if we assume that the density of RyR on SR surface is constant, the number of these ion channels results to be proportional to $\gamma^{n_{v}}$.

Regarding J_3_ flux, we have modeled SERCA pump activity following Lyton et al. [24], and it follows that:

$$J_{3}=c\frac{{\left[Ca^{2+}\right]}_{i}^{n_{s}}}{K_{s}^{n_{s}}+{\left[Ca^{2+}\right]}_{i}^{n_{s}}}$$ (1.5)

with *c* representing the maximum Ca^2+^ flux through SERCA pumps, *K*_*s*_ the corresponding half saturation constant, and *n*_*s*_ a Hill coefficient.

In the cytoplasm, Ca^2+^ ions can be either free or bound to buffering proteins. By definition Ca^2+^ buffer (*β*) is the ratio between the increment in Ca^2+^ bound to proteins and the increment in free [Ca^2+^], so it follows that:

$$\beta =\frac{\Delta {\left[Ca^{2+}\right]}_{B}}{\Delta \left[Ca^{2+}\right]}.$$ (1.6)

Assuming infinitesimal small increments, Eq (1.6) can be solved by Eq (1.7).

$$\beta =\frac{B_{T}K_{D}}{{\left[\left[Ca^{2+}\right]+K_{D}\right]}^{2}},$$ (1.7)

where *B*_*T*_ represents the total amount of Ca^2+^ binding sites with a dissociation constant K_D_.

However, this definition is rather impractical because is very difficult to determine the amount of Ca^2+^ bound to proteins in a cell. However, it is rather easy to determine the total amount of Ca^2+^ added to the cell [21], and then a new Ca^2+^ buffering capacity can be defined as:

$$\beta_{T}=\frac{\Delta {\left[Ca^{2+}\right]}_{T}}{\Delta \left[Ca^{2+}\right]},$$ (1.8)

and in this case, it turns out that Eq (1.8) is related to Eq (1.7) by the following:

$$\beta_{T}=\beta +1.$$ (1.9)

Interestingly, the cytoplasmic Ca^2+^ buffering of smooth muscle and chromaffin cells appears to be constant [21,25]. We have modeled the relation between free and total cytoplasmic [Ca^2+^] as:

$$\Delta {\left[Ca^{2+}\right]}_{i}=\frac{\Delta {\left[Ca^{2+}\right]}_{Ti}}{\beta }.$$ (1.10)

Regarding the RyR open probability, it is known that it increases when Ca^2+^ ions bind this channel on its cytoplasmic side, a process regarded as Ca^2+^ induced Ca^2+^ release (CICR), which functions at low efficiency in smooth muscle cell [26]. In this work we have used caffeine to increase the Ca^2+^ affinity of smooth muscle’s RyR so they open even when Ca^2+^ is at basal level. To account for the above described phenomenon we have assumed that each of the four subunits of RyR has binding sites for Ca^2+^ and caffeine, and these molecules interact cooperatively when they are bound. From this consideration and the assumption of chemical equilibrium, the probability that there is a Ca^2+^ ion bound to its corresponding binding site, regardless of the state of the caffeine site is:

$$\frac{\frac{{\left[Ca^{2+}\right]}_{i}}{K_{C}}+k_{F}\frac{{\left[Ca^{2+}\right]}_{i}}{K_{C}}\frac{\left[Caff\right]}{K_{F}}}{1+\frac{{\left[Ca^{2+}\right]}_{i}}{K_{C}}+\frac{\left[Caff\right]}{K_{F}}+k_{F}\frac{{\left[Ca^{2+}\right]}_{i}}{K_{C}}\frac{\left[Caff\right]}{K_{F}}}=\frac{\frac{{\left[Ca^{2+}\right]}_{i}}{K_{C}}\left(1+k_{F}\frac{\left[Caff\right]}{K_{F}}\right)}{1+\frac{{\left[Ca^{2+}\right]}_{i}}{K_{C}}\left(1+k_{F}\frac{\left[Caff\right]}{K_{F}}\right)+\frac{\left[Caff\right]}{K_{F}}},$$

where *K*_*C*_ is the dissociation constant of RyR-Ca^2+^ complex, *K*_*F*_ is the dissociation constant of the RyR-caffeine complex, and *k*_*F*_ > 1 accounts for the cooperativity between Ca^2+^ and caffeine. Under the assumption that [*Caff*]/*K*_*F*_ ≪ 1, the above expression can be rewritten as:

$$\frac{{\left[Ca^{2+}\right]}_{i}\left(1+k_{f}\left[Caff\right]\right)}{K_{C}+{\left[Ca^{2+}\right]}_{i}\left(1+k_{f}\left[Caff\right]\right)},$$

with *k*_*f*_ = *k*_*F*_ / *K*_*F*_. Finally, considering that Ca^2+^ needs to be bound to all four subunits in order for the channel to open, and that there is a certain amount of cooperativity among subunits, the RyR open probability can be written as [27]:

$$P_{o}\left({\left[Ca^{2+}\right]}_{i},\left[Caff\right]\right)=\frac{{\left[{\left[Ca^{2+}\right]}_{i}\left(1+\left[Caff\right]\right)\right]}^{n_{F}}}{K_{C}^{n_{F}}+{\left[{\left[Ca^{2+}\right]}_{i}\left(1+k_{f}\left[Caff\right]\right)\right]}^{n_{F}}},$$ (1.11)

with *n*_*F*_ being a Hill coefficient. The Ca^2+^ ion in the SR can be either free or bound to proteins.

Let P denote concentration of Ca^2+^ binding sites within the SR, and P_C_ the concentration of sites already occupied with Ca^2+^. Under the assumption of chemical equilibrium, it follows that:

$$K_{R}=\frac{P{\left[Ca^{2+}\right]}_{FSR}}{P_{C}},$$ (1.12)

where *K*_*R*_ is the corresponding dissociation constant of this protein P. Moreover, let P_E_ denote the concentration of Ca^2+^ binding sites in the absence of luminal Ca^2+^. Hence, it follows from the assumption that the total number of Ca^2+^ binding sites remains constant that

$$P=P_{E}-P_{C}.$$ (1.13)

By solving for P_C_ from Eq (1.12) and Eq (1.13) we obtain

$$P_{C}=P_{E}\frac{{\left[Ca^{2+}\right]}_{FSR}}{K_{R}+{\left[Ca^{2+}\right]}_{FSR}},$$ (1.14)

and because Ca^2+^ can only be free or bound then,

$${\left[Ca^{2+}\right]}_{TSR}={\left[Ca^{2+}\right]}_{FSR}+P_{C}.$$ (1.15)

Hence, by solving for [Ca^2+^]_FSR_ from Eq (1.14) and Eq (1.15) we get the following expression for the concentration of free luminal Ca^2+^ in terms of the total Ca^2+^ concentration in the SR:

$${\left[Ca^{2+}\right]}_{FSR}=\frac{1}{2}\xi +\frac{1}{2}\sqrt{\xi^{2}+4{\left[Ca^{2+}\right]}_{TSR}K_{R}},$$ (1.16)

where *ξ* = [*Ca* ^2+^]_*TSR*_ − *P*_*E*_ − *K*_*R*_. This is the classical approach for saturable kinetics (Standard Kinetics, SK) because, as seen in Eq (1.13), the amount of free binding sites is depleted as the amount of luminal Ca^2+^ increases and the available sites are bound. In this case the Ca^2+^ buffer capacity can be estimated by Eq (1.7) (S2A Fig). This graph shows that *β* is a decreasing function of Ca^2+^ that approaches zero when *Ca* ^2+^ >> *K*_*D*_. Nonetheless, this type of kinetics cannot explain phase 1 of Ca^2+^ release observed in smooth muscle [11,16]. We have come to realize that Ca^2+^ binding to calsequestrin shows an increase of B_max_ as Ca^2+^ was elevated (S3 Fig). Based on fitting, previously published data [28], to binding curves with different values of B_max_. We have observed that B_max_ value increases as a function of the window of [Ca^2+^] that was used to calculate the binding curve (S3 Fig). Base on this type of behavior, we have suggested a new kinetic model called **Kinetics on Demand (KonD)**. Additionally, there are data using X-ray microanalysis showing that sections of the ER behave as if the buffering capacity is extremely large [29] in combination with regions in which the free luminal SR [Ca^2+^] does not change, a situation that might reflect the ability of luminal Ca^2+^ buffering proteins to have a KonD type of Ca^2+^ binding. This observation is compatible with the assumption that new Ca^2+^ binding sites become apparent as the already available ones are bound to Ca^2+^. Since there is no mathematical framework to model this type of behavior, we decided to test this new paradigm by assuming that Eq (1.13) can be substituted with the following:

$$P=P_{E}+P_{C}.$$ (1.17)

If we take this new relation and repeat the procedure leading to Eq (1.16) we have obtained the following expression relating the free and the total SR Ca^2+^ concentrations:

$${\left[Ca^{2+}\right]}_{FSR}=\frac{1}{2}\xi -\frac{1}{2}\sqrt{\xi^{2}-4{\left[Ca^{2+}\right]}_{TSR}K_{R}},$$ (1.18)

where *ξ* = [*Ca* ^2+^]_*TSR*_ + *P*_*E*_ + *K*_*R*_.

Interestingly, this new type of kinetics displays a growing Ca^2+^ buffering capacity (S2B Fig), as long as Ca^2+^ is below the dissociation constant because

$$\beta =\frac{B_{T}K_{D}}{{\left[\left[Ca^{2+}\right]-K_{D}\right]}^{2}}.$$ (1.19)

when Ca^2+^ is above *K*_*D*_ then the buffering power is also a decreasing function as it is the case of Standard Kinetics.

### Parameter estimation

The parameters used in the model are shown in Table 1. These were obtained by adjusting data using [Ca^2+^]_i_ responses induced by 20 mM caffeine and by assuming that the amount of SR responding to caffeine varied from cell to cell and was between 1 and 10% of the cell volume.

**Table 1:** Parameters used for KonD mathematical model simulation of variability of [Ca^2+^]_i_ responses to caffeine for control condition (20 mM caffeine), lower caffeine (2 mM) and inhibited SERCA pump with thapsigargin. All parameters values were the same as those obtained with 20mM caffeine-induced [Ca^2+^]_i_ response except where indicated with bold letters. The description of parameters can be found in Supporting Information (S1 Text).

Parameters	20 mM caffeine	2 mM caffeine	Thapsigargin + 20 mM caffeine
a	35 s^-1^	35 s^-1^	35 s^-1^
b	72.2 s^-1^	72.2 s^-1^	72.2 s^-1^
c	11.25 μM/s	11.25 μM/s	11.25 μM/s
[Ca^2+^]_i_	75 nM	75 nM	75 nM
*γ*	1–10%	1–10%	1–10%
n_*v*_	1.7	1.7	1.7
K_s_	300 nM	300 nM	300 nM
n_s_	2	2	2
β	100	100	100
n_F_	1.8	1.8	1.8
k_f_	4 mM^-1^	4 mM^-1^	**0.3 mM** ^**-1**^
[Caffeine]	20 mM	**2 mM**	20 mM
[Ca^2+^]_TSR_	1.65 mM	1.65 mM	**0.75 mM**
[Ca^2+^]_FSR_	150 μM	150 μM	150 μM
K_R_	151.5 μM	151.5 μM	151.5 μM
Thapsigargin	0	0	**1**
Calsequestrin	10	10	**4**

## Results

### Ca^2+^ release from the SR involved four different phases in smooth muscle cells

**Fig 1:**
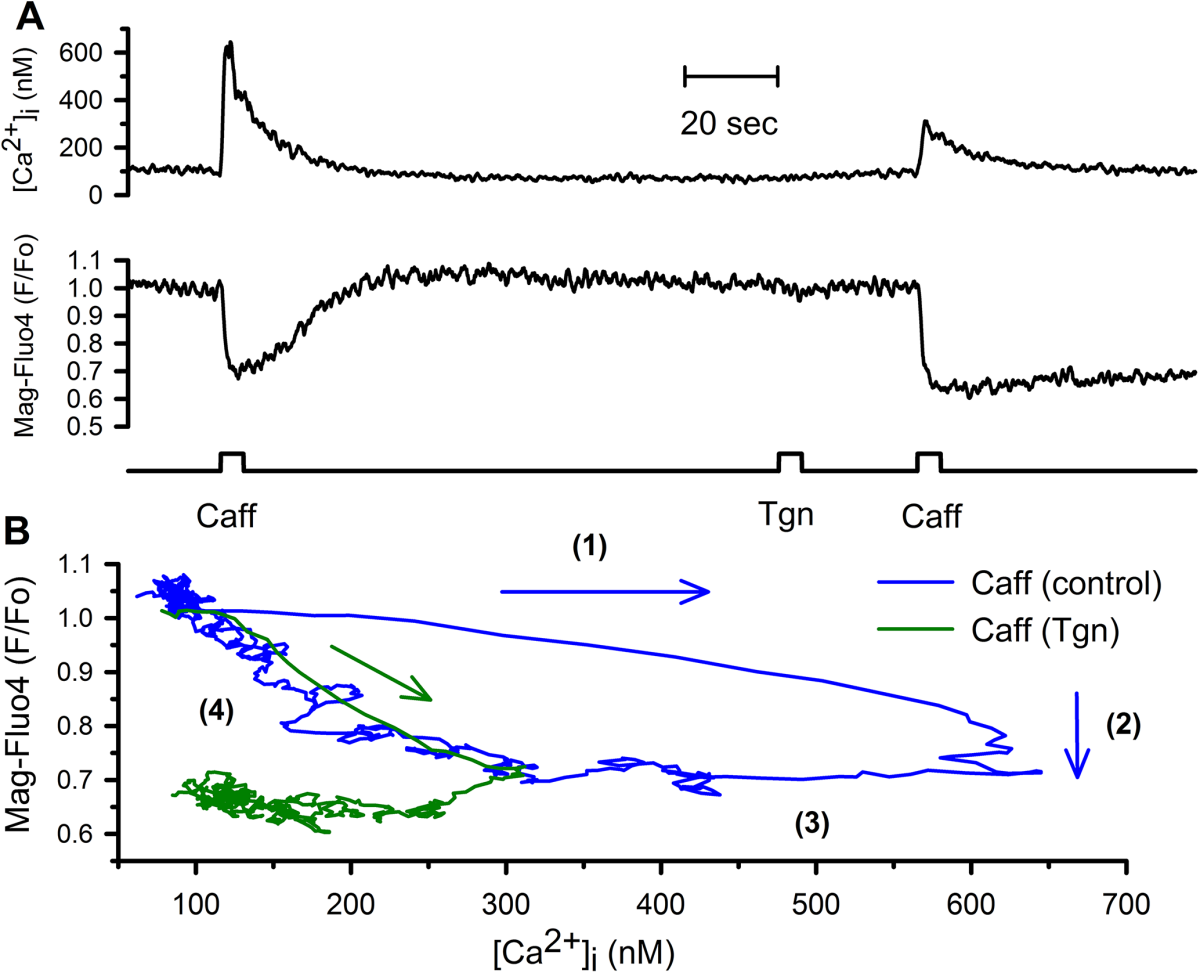
Caffeine-induced Ca^2+^ release involves four phases that require active SERCA pumps in smooth muscle cells. Single freshly isolated smooth muscle cells from guinea pig urinary bladder were loaded with both fura-2 and Mag-Fluo-4 to measure [Ca^2+^]_i_ and [Ca^2+^]_FSR_, respectively. (A) Caffeine application (20 mM with a puffer pipette for 5 seconds) induced a transient increase of the [Ca^2+^]_i_ and a transient reduction of the [Ca^2+^]_FSR_. Inhibition of SERCA pumps with thapsigargin reduced both the amplitude and the rate of [Ca^2+^]_i_ response; however, (B) close examination of these responses show that caffeine induced an increase in the [Ca^2+^]_i_ before any reduction of the [Ca^2+^]_FSR_ (phase 1) followed by a sharp reduction in the [Ca^2+^]_FSR_ without any effect on the [Ca^2+^]_i_ (phase 2). Interestingly, the inhibition of SERCA pumps with thapsigargin fused phase1 and phase 2 in a linear reduction of the [Ca^2+^]_FSR_ associated with a smaller increase of the [Ca^2+^]_i_. Ca^2+^ recording were carried out as previously described (10).

Simultaneous recordings of [Ca^2+^]_i_ and [Ca^2+^]_FSR_ in smooth muscle cells have demonstrated that activation of RyRs by caffeine induces a large increase in [Ca^2+^]_i_ before any reduction of the [Ca^2+^]_FSR_ (phase 1), which is followed by a large reduction of [Ca^2+^]_FSR_ and a plateau of the [Ca^2+^]_i_ increase (phase 2). Additionally, it was found that rapid inhibition of SERCA pump reduces both the amplitude and the rate of rise of the caffeine-induced [Ca^2+^]_i_ response [4,11,16]. Fig 1A shows the effect of 5-sec stimulation with caffeine on the simultaneous recording of the [Ca^2+^]_i_ (upper trace) and the [Ca^2+^]_FSR_ (middle trace) before and after the inhibition of SERCA pumps with thapsigargin. Application with a second puffer pipette of 10 μM thapsigargin for 5 sec was enough to inhibit completely the recovery of the [Ca^2+^]_FSR_ after terminating the second application of caffeine (Fig 1A). This effect agrees with, the already described, irreversible inhibitory action of thapsigargin on SERCA pump activity [4]. Fig 1B blue line indicates those four phases of Ca^2+^ release for data shown in Fig 1A in the absence of thapsigargin. The effect of inhibiting SERCA pumps with thapsigargin on the phase diagram is shown in Fig 1B (green line). Interestingly, phase 1 and 2 are fused together in a single phase showing now a linear correlation between the reduction in the [Ca^2+^]_FSR_ and the diminished increase in the [Ca^2+^]_i_ response.

### Kinetics on demand model fitted the caffeine-induced Ca^2+^ release

**Fig 2:**
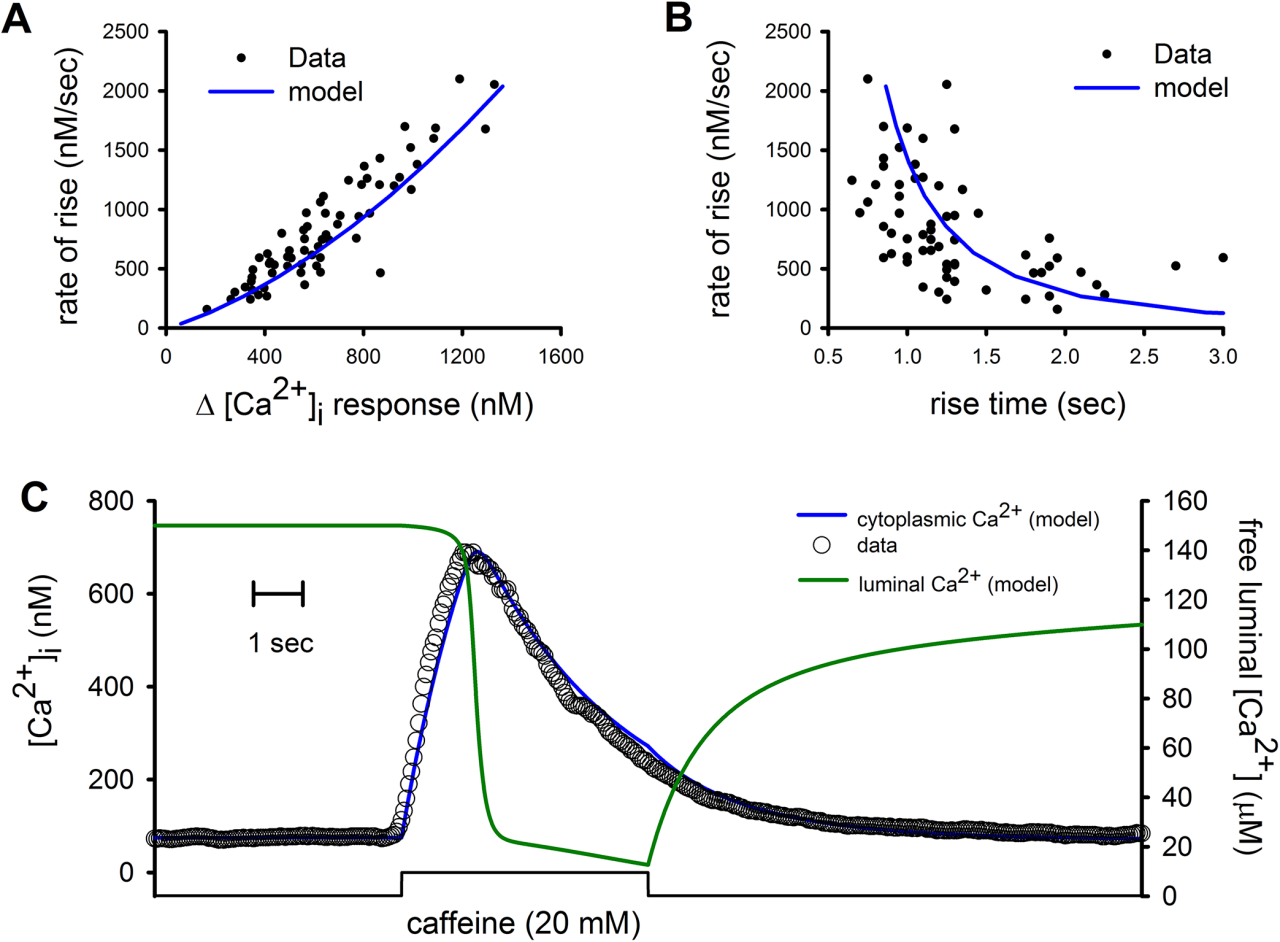
[Ca^2+^]_i_ responses induced by 20 mM caffeine and their fitting by KonD model. (A) Variability of the amplitude and the rate of rise of caffeine-induced [Ca^2+^]_i_ responses for 66 different cells and the fitting by KonD model (blue line) of these responses. (B) Same responses as shown in (A) but comparing the time to peak and the rate of rise that was fitted by the model. (C) Time course of the [Ca^2+^]_i_ response (open circles) fitted by the model (blue line) and the reduction in [Ca^2+^]_FSR_ predicted by the KonD model (green line).

To get an insight on how SERCA pumps might control SR Ca^2+^ release in smooth muscle cells we have developed a simple deterministic mathematical model based on the [Ca^2+^]_i_ response to two different caffeine concentrations (2 and 20 mM) and the effect of rapid inhibition of SERCA pump with thapsigargin on the 20 mM caffeine-induced [Ca^2+^]_i_ response. The mathematical model is based on a new concept named Kinetics-on-Demand (KonD), where Ca^2+^-binding sites of SR Ca^2+^-binding proteins are an increasing function of [Ca^2+^] (see Model development). RyRs were modeled as homotetrameric channels activated by Ca^2+^ and caffeine. Application of 20 mM caffeine with a puffer pipette to freshly isolated smooth muscle cells loaded with Fura-2 produced a transient [Ca^2+^]_i_ response with large variability in the amplitude, the rate of rise and the time to peak. Our mathematical model was able to reproduce this variability by assuming that the SR volume responding to caffeine varied between 1 and 10% of cell volume. The values for the rest of the parameters are shown in Table 1 and were determined using those that fitted [Ca^2+^]_i_ responses induced by 20 mM caffeine. Fig 2A shows the relationship between the amplitude of 20 mM caffeine-induced [Ca^2+^]_i_ responses and the corresponding maximal rate of rise for 66 different cells and how the model fitted this relationship by varying the fraction of responding SR volume (blue line). The KonD model was also able to reproduce the relation between the peak rate of rise and the rise time of the caffeine-induced [Ca^2+^]_i_ responses (Fig 2B). This model was used also to simulate the time course of caffeine-induced [Ca^2+^]_i_ response (Fig 2C, blue line) together with the changes in the [Ca^2+^]_FSR_ (Fig 2C, green line).

**Fig 3:**
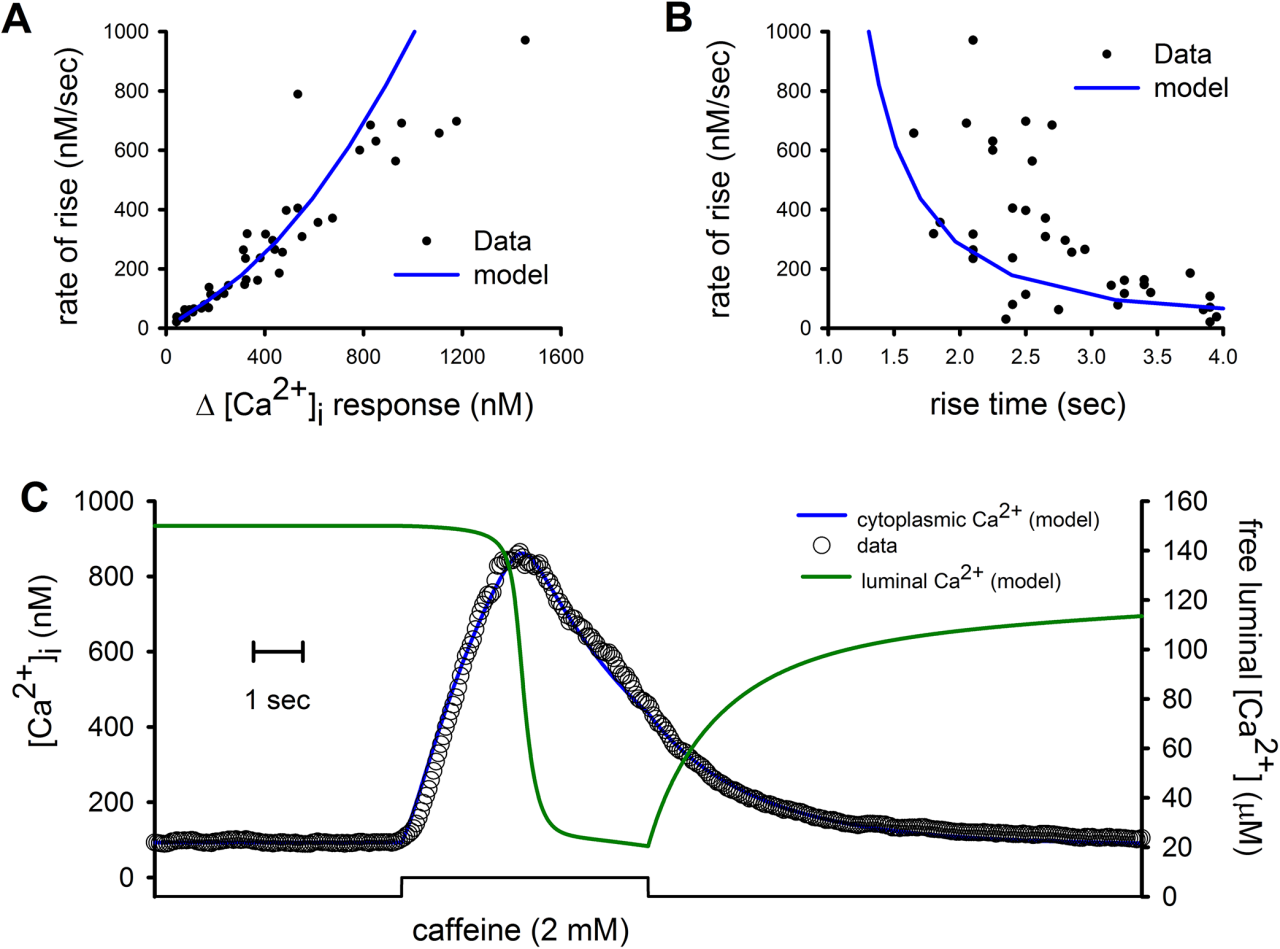
[Ca^2+^]_i_ responses induced by 2 mM caffeine and their fitting by KonD model. (A) Variability of the amplitude and rate of rise of the caffeine-induced [Ca^2+^]_i_ responses for 49 different cells and the fitting by KonD model (blue line) of these responses. (B) Comparison of the rise time vs rate of rise for the same [Ca^2+^]_i_ responses and fitting by KonD model (blue line). (C) Time course for both [Ca^2+^]_i_ and KonD model (blue line) together with the [Ca^2+^]_FSR_ response derived from the model (green line).

To assess the robustness of our mathematical model, we used the parameters found for fitting 20 mM-caffeine induced [Ca^2+^]_i_ responses to model the responses obtained with 2 mM caffeine. In this case the same parameters shown in Table 1 were used, except for the concentration of caffeine. The application of 2 mM caffeine produced on average smaller and slower [Ca^2+^]_i_ responses that were reproduced by the KonD model. There were also good fittings between the maximal rate of [Ca^2+^]_i_ rise and either the peak [Ca^2+^]_i_ response (Fig 3A) or the rise time (Fig 3B). These parameters also fitted the time course of the 2 mM caffeine-induced [Ca^2+^]_i_ response (Fig 3C, blue line) and show the time-course for the reduction of the [Ca^2+^]_FSR_ (Fig 3C, green line). These results with 2 mM caffeine suggest that the KonD model is robust enough to evaluate which parameters play a role in the SERCA-inhibited [Ca^2+^]_i_ responses induced by caffeine.

### Inhibition of SERCA Ca^2+^ pumps changed caffeine-induced Ca^2+^ release from KonD model to SK model

**Fig 4:**
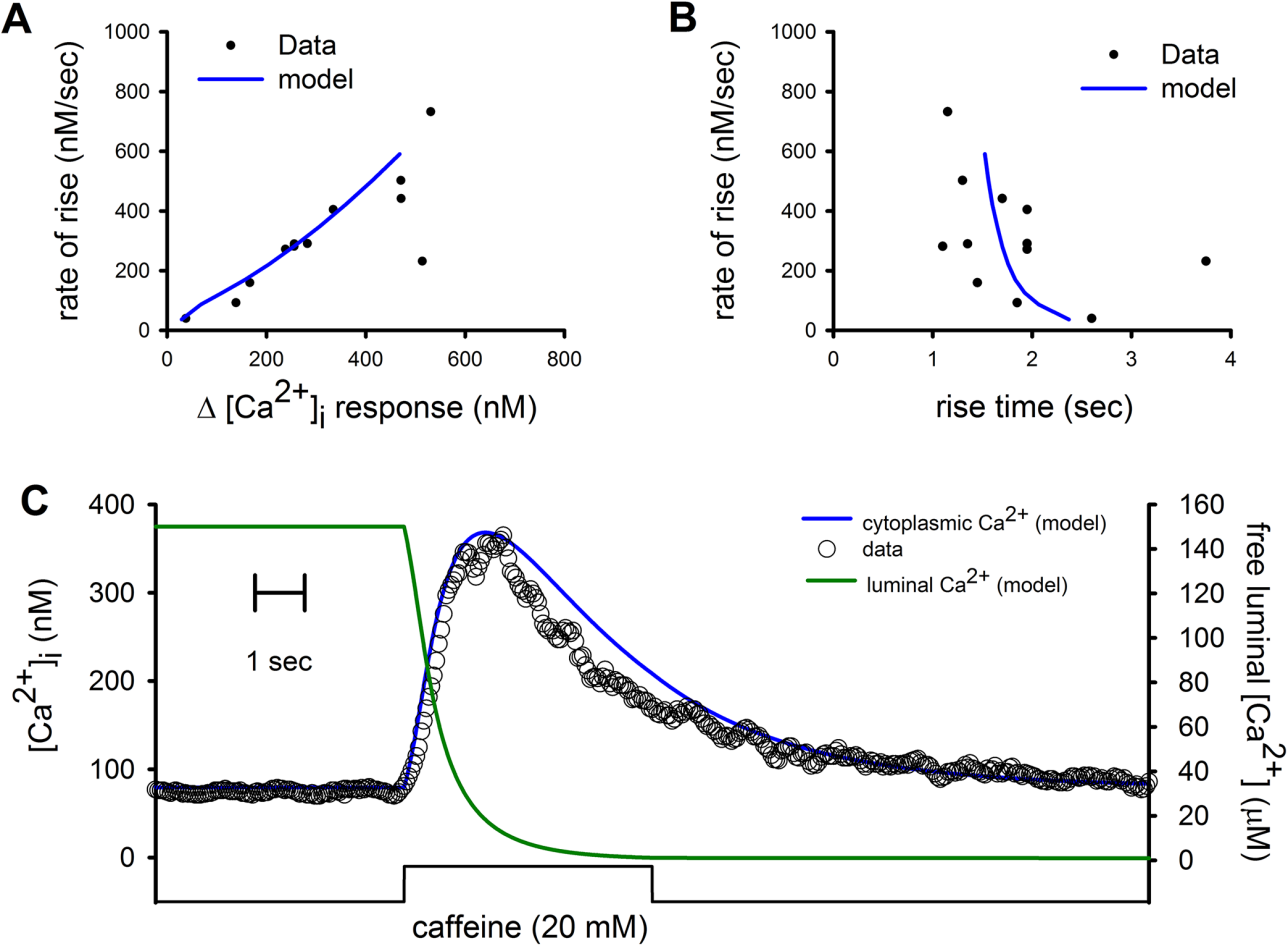
Effect of SERCA pump inhibition by thapsigargin on 20 mM caffeine-induced [Ca^2+^]_i_ responses. (A) [Ca^2+^]_i_ responses of 12 different cells that were exposed to thapsigargin for 5 seconds, 30 seconds before the application of caffeine, were smaller in amplitude and slower and (B) with a larger rise time. The mathematical model fitted this relationship but only after switching from KonD model to SK model (blue lines). (C) Time course of the [Ca^2+^]_i_ response to 20 mM caffeine from a cell that had been previously exposed to thapsigargin together with fitting by modified SK model (blue line) and the resulting reduction in the [Ca^2+^]_SR_ from this model (green line). Notice there was no recovery of the [Ca^2+^]_FSR_ as expected for inhibited SERCA pumps.

Application of thapsigargin (10 μM) with a second puffer pipette for 5 seconds and 30 seconds before the application of caffeine resulted, as expected, in smaller (Fig 4A) and slower (Fig 4B) [Ca^2+^]_i_ responses. In this case the reduction in the [Ca^2+^]_FSR_ occurred at a higher rate than when SERCA pumps are active and yet, the [Ca^2+^]_i_ response was both smaller and slower (Fig 4C, green line).

**Fig 5:**
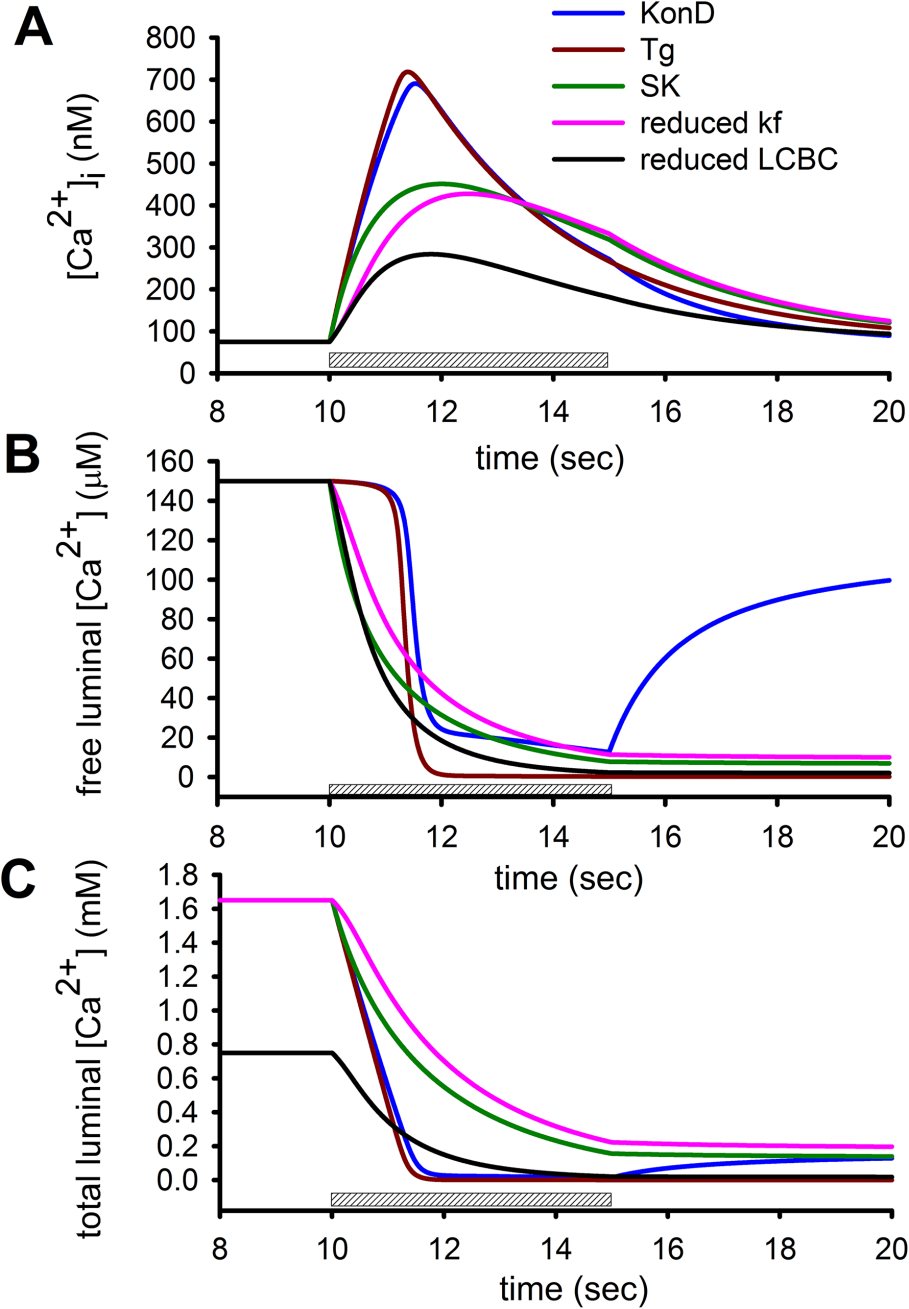
Inhibition of SERCA pump switches Ca^2+^ release from KonD condition to SK situation. The control response to application of 20 mM caffeine using KonD model is shown for (A) [Ca^2+^]_i_, (B) [Ca^2+^]_FSR_ and (C) [Ca^2+^]_TSR_ (blue lines). Inhibition of SERCA pump with thapsigargin (B, C, red line) impaired recovery of [Ca^2+^]_FSR_ but did not alter the [Ca^2+^]_i_ response (A, red line). Switching from KonD to SK significantly reduced the amplitude of the [Ca^2+^]_i_ (A, green line) because decreased the reduction of [Ca^2+^]_TSR_ (C, green line) although accelerated the reduction of the [Ca^2+^]_FSR_ (B, green line). To reduce both the amplitude and the rate of rise, it was reduced the number of release channels involved (*k*_*f*_, magenta line) and accordingly we have to reduce also the amount of luminal Ca^2+^ binding proteins involved (LCBC, solid line). Caffeine was present for a period of 5 seconds to activate RyRs beginning at the time point of 10 seconds as indicated by the dashed bar.

The phase diagram displayed in Fig 1B shows that inhibition of SERCA pump combined phases 1 and 2 resulting now in a linear relation between the increase in the [Ca^2+^]_i_ and the reduction of the [Ca^2+^]_FSR_. We have used KonD model to determine which parameters need to be changed to reproduce the effect of thapsigargin. This exercise is shown in Fig 5. Where Fig 5A is the [Ca^2+^]_i_ responses to the application of 20 mM caffeine where indicated, while Fig 5B corresponds to the [Ca^2+^]_FSR_ and Fig 5C shows the total SR [Ca^2+^] ([Ca^2+^]_TSR_) which corresponds to the sum of both [Ca^2+^]_BSR_ and [Ca^2+^]_FSR_. The complete inhibition of SERCA pump by itself did not importantly alter (red line) the [Ca^2+^]_i_ response to the application of 20 mM caffeine (Fig 5A, blue line) but as expected, completely eliminated the recovery of [Ca^2+^]_FSR_ after caffeine application (Fig 5B, red line). However, we observed a significant reduction in the caffeine-induced [Ca^2+^]_i_ response by changing the way that luminal Ca^2+^-binding proteins interact with Ca^2+^ by using now SK condition instead of KonD (Fig 5A, green line). Interestingly, the main difference is that the delay for the reduction in the [Ca^2+^]_FSR_ has disappeared (Fig 5B, green line) and although the total amount of Ca^2+^ released by the SR is the same in both conditions; in the SK condition occurs at a slower rate than at KonD (Fig 5C, green line vs blue line). This slower kinetics of Ca^2+^ release increased the effect of cytoplasmic Ca^2+^ removal mechanisms on the [Ca^2+^]_i_ response. The effect of thapsigargin on caffeine-induced [Ca^2+^]_i_ response is a reduction of both the peak amplitude and the rate of rise. While the former was accomplished by switching from KonD to SK, the latter required a reduction of both *k*_*f*_ parameter (see Model development) and the amount of luminal Ca^2+^ binding proteins involved during Ca^2+^ release (Fig 5A, solid line). Since the time taken by thapsigargin to produce these two effects is rather short, we think that the concentration of luminal Ca^2+^ binding proteins is not modified and instead there is a reduction in the number of RyRs that are coupled to the luminal Ca^2+^-binding proteins and responding to caffeine. This situation effectively reduces the amplitude of the caffeine-induced [Ca^2+^]_i_ response without any reduction of the [Ca^2+^]_TSR_. However, to achieve this effect in our model we decided to reduce the [Ca^2+^]_TSR_ to fit the recorded data (Fig 5C solid line).

**Fig 6:**
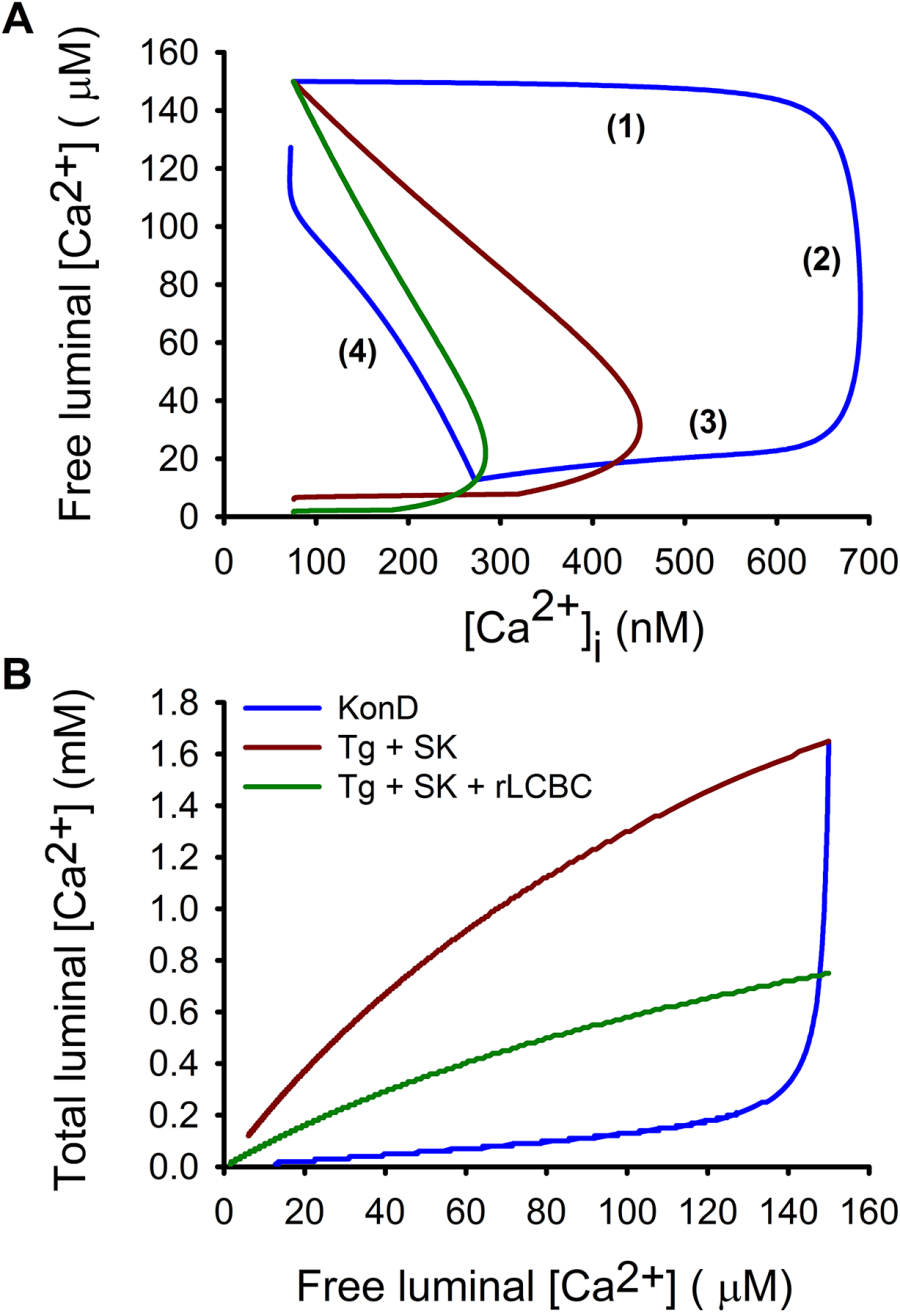
KonD model reproduces phase diagram of Ca^2+^ release. (A) KonD model reproduces phase diagram seen in caffeine-induced Ca^2+^ release (blue line) and this is altered by inhibiting SERCA pump and switching to SK condition (red line). Reducing the number of RyRs participating and the amount of luminal Ca^2+^-binding proteins (LCBC) reduces even further the amplitude of the [Ca^2+^]_i_ response but with a similar phase diagram (green line) that compares very well with experimental data (Fig 1). (B) KonD condition generated a luminal Ca^2+^ buffering power curve with a positive curvature as has been observed in skeletal muscle (blue line). This explains a large supply of Ca^2+^ to the cytoplasm (due to a large reduction in the [Ca^2+^]_TSR_ with minimal reduction of the [Ca^2+^]_FSR_). Switching to SK condition generated a negative curvature that means a larger reduction in the [Ca^2+^]_FSR_ but a much smaller reduction of the [Ca^2+^]_TSR_ (red line) and accordingly a smaller increase in the [Ca^2+^]_i_. Reducing the amount of luminal SR Ca^2+^ binding protein participating in response to caffeine decreased the total amount of Ca^2+^ supplied by SR (green line).

The KonD model can reproduce the four phases of Ca^2+^ release, particularly the transition from phase 1 to phase 2 (Fig 6A, blue line). Importantly, switching from KonD to SK showed that phase 1 and 2 disappeared and resulted now in a linear relationship between the [Ca^2+^]_FSR_ and the [Ca^2+^]_i_ (Fig 6A, red line). Comparison between the [Ca^2+^]_FSR_ and [Ca^2+^]_TSR_ clarifies why KonD condition is more efficient in increasing [Ca^2+^]_i_ than SK. In KonD the Ca^2+^ supplied to the cytoplasm comes mainly from Ca^2+^ bound to proteins with minimal reduction in the [Ca^2+^]_FSR_ (Fig 6B, blue line) while in the SK situation happens exactly the opposite (Fig 6B, red line). This is very important because explains why the [Ca^2+^]_FSR_ does not correlate with the supply of Ca^2+^ to the cytoplasm. Additionally, our mathematical model suggests that SERCA pump modulates the number of release channels coupled to luminal Ca^2+^ binding proteins, such that inhibition of SERCA can produce also a reduction of the amplitude of Ca^2+^ response by decreasing the number of luminal Ca^2+^ binding proteins participating in Ca^2+^ release response (Fig 6B, green line).

### KonD model explained the refractory period associated with the recovery of [Ca^2+^]_FSR_ after caffeine-induced Ca^2+^ release

**Fig 7:**
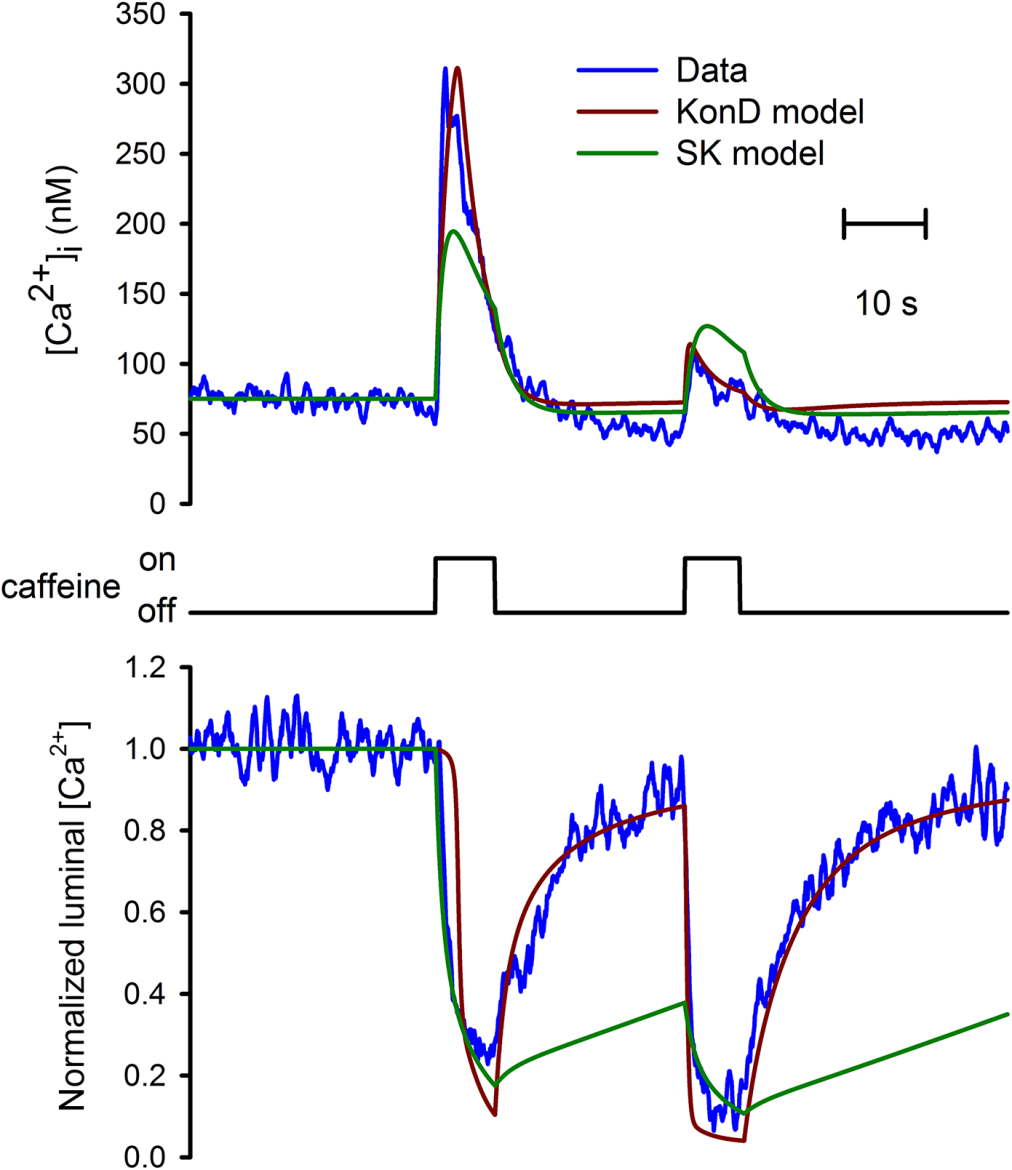
Refractory [Ca^2+^]_i_ response to the application of caffeine. A set of experimental results (blue line) of simultaneous recording of [Ca^2+^]_i_ (upper trace) and SR Ca^2+^ levels (bottom trace) in response to the application of two pulses of caffeine (middle trace) are presented. Notice that by 30 s after the first application of caffeine both [Ca^2+^]_i_ and SR Ca^2+^ levels have recovered to resting levels. A second application of caffeine produce the same response in the SR Ca^2+^ with minimal effect on the [Ca^2+^]_i_. The numerical simulations of KonD model (red line) and SK model (green line) are also shown. The simulation for both models was calculated by solving numerically the differential equations with the parameters shown in Table 1 for 20mM of caffeine with parameter gamma equals to 2.54%. Changing parameter gamma helps SK model to fit the caffeine-induced [Ca^2+^]_i_ transient but does not show the same time course for the recovery of the [Ca^2+^]_FSR_ (S4 Fig).

The activation of ryanodine receptors by a saturating concentration of caffeine produced a transient increase in the [Ca^2+^]_i_ due to Ca^2+^ release from the SR. The application of a second pulse of caffeine just 30 seconds after the first pulse, produced a significantly smaller transient [Ca^2+^]_i_ response of only 20% in amplitude [21]. A straightforward explanation would be that the SR Ca^2+^ store did not have enough time to recover after caffeine-induced Ca^2+^ release. However, the recorded [Ca^2+^]_FSR_ shows that this time of 30 seconds was enough to reach the initial level of the [Ca^2+^]_FSR_ [4,11]. This is a paradox because the SK condition says that recovery of the free luminal [Ca^2+^] implies the complete recovery of total SR [Ca^2+^], which in turn should generate a [Ca^2+^]_i_ response of similar amplitude to the one produced by the first application of caffeine. Fig 7 shows the fitting by either the KonD model (red line) or the SK model (green line) of the simultaneous recording (blue line) of both the [Ca^2+^]_i_ (upper traces) and the changes in the free luminal SR [Ca^2+^] (lower traces) in response to the dual application of saturating concentrations of caffeine (middle trace). The parameters used here are exactly the same to those shown in Table 1 and employed for fitting the response to 20 mM caffeine shown in Fig 2 except, that here, the ratio between the SR and the cytoplasm was modify to fit the amplitude of the caffeine-induced [Ca^2+^]_i_ response. The same parameters were used for the SK model. Since caffeine released the same amount of Ca^2+^ from the SR for both models, the reduced amplitude of the [Ca^2+^]_i_ response seen with the standard model was due to the competition for Ca^2+^ between the luminal SR Ca^2+^ binding proteins and the release channels, a situation that is not observed in the KonD model as demonstrated in Fig 6. Since the SK model does not have phase 1, the reduction in the luminal SR [Ca^2+^] slightly precedes the recorded reduction of the luminal SR [Ca^2+^]. On the other hand, KonD model shows an exaggerated phase 1. Interestingly, the KonD model shows a rapid recovery of the free luminal SR [Ca^2+^] that does not involve the recovery of the total SR [Ca^2+^] (see Fig 5) while in the SK model, since these two parameters go together; there is only a minimal recovery of the [Ca^2+^]_FSR_. This result is also true even when using parameters that best fit the caffeine-induced [Ca^2+^]_i_ response (S4 Fig). This discrepancy between these two models, allows KonD model to explain the apparent paradox of having a recovered free luminal SR [Ca^2+^] with a still reduced total SR [Ca^2+^]. It appears then that KonD describes much better how the SR luminal Ca^2+^ binding proteins behave during caffeine-induced Ca^2+^ release in smooth muscle cells.

## Discussion

### New Ca^2+^ binding kinetic model based on how calsequestrin binds Ca^2+^

A deterministic mathematical model has been developed to simulate Ca^2+^ release from the SR and the role of SERCA pump in facilitating this release. The main contribution of this model is the realization that experimental data cannot be fitted using the SK condition for Ca^2+^ binding to proteins. We have assumed a new type of kinetics, KonD, where the number of Ca^2+^ binding sites increases in response to the binding of Ca^2+^ and the opposite happens when there is a reduction of the [Ca^2+^] (S3 Fig). This idea is supported by the work done with calsequestrin [15], where our own analysis of their Ca^2+^ binding data suggest that B_max_ (the total amount of Ca^2+^ binding sites) increases as [Ca^2+^] is increased, i.e. B_max_ = 22 in the range of 0 to 0.5 mM [Ca^2+^], and goes to 40 between 0.5 and 3 mM [Ca^2+^], B_max_ increases to 65 for the range of 3 to 7 mM [Ca^2+^] and increases again to 79 from 7 to 20 mM [Ca^2+^] (see Supporting Information). Moreover, it has been shown that this increase in B_max_ requires protein oligomerization because using a calsequestrin mutant incapable of aggregation shows a saturable constant B_max_ of 22 all the way to 20 mM [Ca^2+^] [15] (S2 Fig) Additionally, the use of energy-dispersive X-ray microanalysis to determine Ca^2+^ storage in the ER of pyramidal neurons show that certain regions of the ER can accumulate Ca^2+^ in the excess of 100 mM/Kg of dry weight, more importantly, these regions are in continuity with regions of the ER that do not accumulate Ca^2+^, this implies that the buffering regions of the ER are not in equilibrium with the non-buffering regions of the ER since the former can accumulate large amounts of Ca^2+^ without any change in the total amount of Ca^2+^ of the nearby non-buffering regions of the ER [29].

### Caffeine-induced Ca^2+^ release by smooth muscle involves four phases that are reproduced by KonD model

One key element that the model needed to reproduce is the phase 1 observed during SR Ca^2+^ release and the transition from phase1 to phase 2. Phase 1 represents a condition of a very high Ca^2+^ buffer capacity since there are large changes in the [Ca^2+^]_i_ with non-existent (particularly for IP_3_Rs, [11]) or minimal reduction in the [Ca^2+^]_FSR_ which is followed by phase 2 characterized by an abrupt reduction of the luminal Ca^2+^ buffering capacity, because there is a large reduction of the [Ca^2+^]_FSR_ with a minimal effect on the [Ca^2+^]_i_. These two phases and the transition between them cannot be reproduced using SK condition of Ca^2+^ binding proteins, i.e. a condition where there is a fixed number of Ca^2+^ binding sites that reach saturation on increasing the [Ca^2+^]. Interestingly, in skeletal muscle, a transition between phase 1 and phase 2 also produces an SR titration curve with a positive curvature instead of the negative curvature that is expected from the SK condition [30]. However, in this situation it has been argued that there is an increase of the Ca^2+^ binding cooperativity of the luminal SR proteins, which produces positive curvature for the relationship between the [Ca^2+^]_FSR_ and the [Ca^2+^]_TSR._ The main disadvantage of this solution is that the higher the Hill coefficients, the smaller the window where the luminal SR Ca^2+^ binding proteins can effectively buffer the luminal Ca^2+^. This goes in the opposite direction to what has been described for both SR and ER in the sense that they have a large Ca^2+^ buffering capacity [29]. Accordingly, our KonD condition can explain both an increasing Ca^2+^ buffer capacity and an SR Ca^2+^ buffering curve with a positive curvature. Both situations have been observed experimentally [11,16,30]. A very important difference between SK situation and KonD condition that becomes evident in Fig 6, is that in SK the reduction of the [Ca^2+^] increases the competition for free Ca^2+^ between the empty Ca^2+^ binding sites of the luminal proteins and the release channel, while this is not the case with the KonD condition because there is an obligatory reduction in the number of Ca^2+^ binding sites due to the reduction of the [Ca^2+^]_FSR_. Additionally, this situation also explains why phase 1 is characterized by a large increase in the [Ca^2+^]_i_ with a minimal reduction of the [Ca^2+^]_FSR_. Actually, in some cases it has been observed that elevation of the [Ca^2+^]_i_, due to Ca^2+^ release from internal stores, is associated with an increase in the luminal [Ca^2+^] [6,11,31,32]. Importantly, this is basically impossible to achieve with the SK condition, but the KonD model will work, provided that the number of release channels that are open is not too large. Indeed, Ikemoto described how the activation of few RyRs in SR microsomes of skeletal muscle by low concentrations of polylysine produces a transient increase in the luminal SR [Ca^2+^] and that this effect is not seen when using saturating concentrations of polylysine [6]. This effect was due to the presence of calsequestrin associated to the membrane of the microsomes [26]. Likewise, we have observed that low concentrations of heparin, to partially inhibit IP_3_Rs, induced a transient increase in the luminal [Ca^2+^]_FSR_ in response to the activation of muscarinic receptors in the same type of smooth muscle cell used in this work [11]. Interestingly, this partial inhibition of IP_3_Rs resulted in larger, but short-lived, [Ca^2+^]_i_ responses to carbachol in agreement with the situation that the driving force for Ca^2+^ release was larger due to a higher luminal [Ca^2+^]_SR_ but involved a lower number of IP_3_Rs [11]. Later on, it has been shown that spontaneous, slow Ca^2+^ waves in heart cells, unexpectedly associate with a simultaneous elevation of the [Ca^2+^]_FSR;_ while the coordinated activation by membrane depolarization of a larger number of RyRs shows the expected reduction of the [Ca^2+^]_FSR_ and the increase of the [Ca^2+^]_i_ [32]. We think that this characteristic of RyRs and the fact that some RyRs are not associated with SR luminal Ca^2+^ binding proteins might explain why KonD model produces an exaggerated phase 1 for caffeine-induced Ca^2+^ release. Moreover, stimulation of Ca^2+^ release by activation of purinergic receptors in globet cells showed a clear elevation of the luminal ER [Ca^2+^] before any increase in the [Ca^2+^]_i_ [31]. In this case it was suggested that K^+^ plays a key role as a counterion in Ca^2+^ release, a situation that has been already demonstrated since the Ca^2+^ release is strongly inhibited in the absence of K^+^ [33]. Interestingly, HeLa cells incubated in the absence of external [Ca^2+^] show a similar amplitude of the histamine-induced [Ca^2+^]_i_ response whether the free luminal ER [Ca^2+^] was either high or low [19]. These data suggest that release channels have access to a Ca^2+^ source that is different to the free luminal [Ca^2+^], most likely the Ca^2+^ bound to luminal ER Ca^2+^ binding proteins. Since globet and HeLa cells do not express calsequestrin, these observations also imply that there are other luminal Ca^2+^ binding proteins that function similarly to calsequestrin but in the ER. Actually, the double KO of calsequestrin did not inhibit Ca^2+^ release in skeletal muscle [34].

### KonD model explains the refractory period of Ca^2+^ release despite normal luminal SR Ca^2+^ level

We have noticed that KonD model predicts a fast recovery of luminal [Ca^2+^]_FSR_ and a much slower recovery of the [Ca^2+^]_TSR_ (compare solid line of Fig 5B versus 5C and see Fig 7). This establishes a refractory period where there is recovery of [Ca^2+^]_FSR_ but not of the [Ca^2+^]_TSR_ so a second [Ca^2+^]_i_ response would be greatly diminished [4,11,21,35]. The opposite situation might happen where [Ca^2+^]_FSR_ can be reduced by leak but not the Ca^2+^ bound to proteins [18].

It has been suggested that Ca^2+^ release from the luminal proteins is more an “active” process than a “passive” one [6]. In other words, SK condition (which is a passive process) cannot explain the transient increase in the luminal [Ca^2+^]. Additionally, Ikemoto proposes that Ca^2+^ release involves 3 stages: 1) conformational activation of release channels, 2) induction of Ca^2+^ release from the luminal proteins and the subsequent increase in the luminal [Ca^2+^] and 3) opening of release channels and Ca^2+^ moving from the SR to the cytoplasm [5–7,36]. In this regard, it has been described that a conserved glutamate in both RyRs and IP_3_Rs can function as a luminal Ca^2+^ sensor, where the increase in the luminal [Ca^2+^] would trigger the opening of the release channel and allow Ca^2+^ movement to the cytoplasm [37].

### Role of SERCA pumps in facilitating Ca^2+^ release

Inhibition of SERCA pumps reduces Ca^2+^ release and in general it has been assumed that this is due to depletion of the internal Ca^2+^ stores. However, this is not always the case, in pancreatic acinar cells the inhibition of SERCA pumps resulted in a slower Ca^2+^ release and disappearance of the Ca^2+^ gradients by a mechanism that does not involve the reduction of the luminal ER [Ca^2+^] [8]. Additionally, SERCA pump activity potentiates histamine-induced Ca^2+^ release in HeLa cells [10]. Rapid inhibition of SERCA pump in heart cells results in a slower velocity of the Ca^2+^ wave without any reduction in the caffeine-sensitive store [9]. The opposite is also true, increased activity of SERCA pump due to overexpression of adrenergic receptors in heart cells results in higher and faster Ca^2+^ release in the absence of an increased loading of SR Ca^2+^ store [14]. SERCA pump inhibition in guinea pig urinary bladder smooth muscle cells results in a decreased amplitude and slower Ca^2+^ release for both RyRs and IP_3_Rs [4], this was the case even when there is no reduction of the [Ca^2+^]_FSR_ [4,11]. Collectively, these data suggest that inhibition of SERCA pump slows Ca^2+^ release while its activation has the opposite effect. However, there is no clear explanation for this effect of SERCA pump. Our mathematical model suggest that inhibition of SERCA pump changes Ca^2+^ release from KonD condition to the less efficient SK situation, so this might explain the slower Ca^2+^ release. Additionally, it appears that there is also a reduction in the number of the release channels participating (k_f_), so this might be the reason behind a lower amplitude of the [Ca^2+^]_i_ transient.

### Alternative models to KonD conditions

KonD condition is the simplest solution, we were able to visualize for an SR Ca^2+^ buffering capacity with a positive curvature; however, although likely in the right direction might not be the right solution. This is because another characteristic of SR Ca^2+^ stores is that Ca^2+^ freely diffuses in the lumen, a condition that is not expected from the KonD condition. The most likely solution is that the trapping of Ca^2+^ by luminal SR Ca^2+^ binding proteins does not occur instantaneously because it depends on the conformational changes and aggregation of proteins, a situation that might take a longer time than the one required for free diffusion of Ca^2+^ in the lumen of SR, so KonD model should consider that the kinetics of dissociation of Ca^2+^ from luminal SR proteins is rather fast while the kinetics of association is rather slow.

A mathematical model of Ca^2+^ spark termination in heart cells has contemplated Ca^2+^ diffusion inside the SR [38,39]. Interestingly, this model says that Ca^2+^ diffusion coefficient in the non-junctional SR is 0.6 x 10^−10^ m^2^ s^-1^ (as previously estimated, [40]) but considers that this coefficient is 5-fold larger in junctional SR, that is 3.5 x 10^−10^ m^2^ s^-1^ [38,39]. However, this contrasts with the concentration of non-mobile calsequestrin. The model says that calsequestrin in the non-junctional SR is 6 mM (as previously estimated, [41]) while it says is 5-fold higher in the junctional SR (30 mM [38,39]). This situation is rather paradoxical because we think that a higher concentration of a non-mobile calsequestrin should become a barrier for Ca^2+^ diffusion. However, this apparent paradox stresses the importance of limiting the competition between calsequestrin and RyRs for free luminal Ca^2+^ to achieve an efficient Ca^2+^ release event. Here, we have obtained the same result by reducing the number of luminal Ca^2+^ binding sites driven by a reduction of the luminal SR [Ca^2+^]. Alternatively, our results can be reproduced if the luminal Ca^2+^ binding proteins diffuse away from the RyRs during the opening of release channels. However, we think that this scenario is unlikely since there is no driving force for this to happen and this process might not be fast enough to avoid competition with RyRs for the free luminal Ca^2+^. Another scenario involves restricted diffusion of both Ca^2+^ and Mag-Fluo-4 (they are maintained in the bulk of the SR away from the RyRs), so they are not in rapid equilibrium with the calsequestrin, which is known to be associated with RyRs [42]. If the diffusion of Ca^2+^ between these two compartments is very low, then it is expected to see recovery of the free luminal SR Ca^2+^ level (provided that SERCA pumps are located where Mag-Fluo-4 is) even when the RyRs are still open (a situation that have not been observed). Alternatively, if the diffusion barrier is not that important, then the transition between phase 1 and phase 2 will not be clear. A situation that was observed only in the presence of thapsigargin. In conclusion, we think that our mathematical model stresses the importance of Ca^2+^ binding proteins rapidly switching from high to low Ca^2+^ buffering capacity during Ca^2+^ release event, and this provides enough free luminal Ca^2+^ for an efficient release by RyRs. How exactly this process is achieved inside the SR is not clear and deserves further studies.

## Supporting Information

S1 Fig Schematic representation. Graphical model of smooth muscle, not at scale, of the two intracellular compartments of interest, as well as those Ca^2+^ fluxes between these two compartments.
(TIF)

S2 Fig Buffer capacity as a function of [Ca^2+^]. (A) Typical relationship for a 3 mM Ca^2+^ binding protein with a K_D_ of 151.1*μM* using Eq (1.7). (B) Ca^2+^ buffering capacity for the same [Ca^2+^] as in A but using KonD model and Eq (1.19) with values indicated in the text.
(TIF)

S3 Fig Fitting of different Ca^2+^ saturation levels of calsequestrin. Models of Ca^2+^ bound to calsequestrin as a function of [Ca^2+^]; data replotted from Fig 5 of reference [28]. Data were fitted to a Hill equation. Fitting results suggests that the maximal number of Ca^2+^ binding sites (B_max_) is increasing as a function of the [Ca^2+^]. The inset shows the fitting for [Ca^2+^] in the range of 0–0.6mM. Park et al. have shown that blocking polymerization of calsequestrin inhibits this effect of increasing B_max_, so B_max_ value stays around 22 for the whole range of [Ca^2+^] tested (triangle line) [28]. At least 5 points were used to calculate B_max_ value higher than 70.
(TIF)

S4 Fig SK model cannot follow the recovery of the free luminal SR [Ca^2+^]. In this case the gamma value used in the SK model (red line) was modified to fit the caffeine-induced [Ca^2+^]_i_ response (blue line). Although there is now a good fitting of the [Ca^2+^]_i_ responses induced by caffeine, the time course of recovery of the [Ca^2+^]_FSR_ is much slower in the SK model than is recorded by the Mag-Fluo-4 indicator. This can be explained by considering that free and total Ca^2+^ go together in the SK model, while in the KonD model (see Figs 5 and 7) there is a rapid recovery of the [Ca^2+^]_FSR_ that precedes the recovery of the [Ca^2+^]_TSR_, which explains the presence of a refractory period due to the recovery of the [Ca^2+^]_FSR_ but not of the total, so the second caffeine-induced Ca^2+^ response is much smaller.
(TIF)

S1 Text Parameter estimation. (DOCX)
