# Supplementary figures and images for: Kinetics on Demand Is a Simple Mathematical Solution that Fits Recorded Caffeine-Induced Luminal SR Ca2+ Changes in Smooth Muscle Cells
Source: PLoS One. 2015 Sep 21;10(9):e0138195. doi: 10.1371/journal.pone.0138195 (PMC4577101; doi:10.1371/journal.pone.0138195)

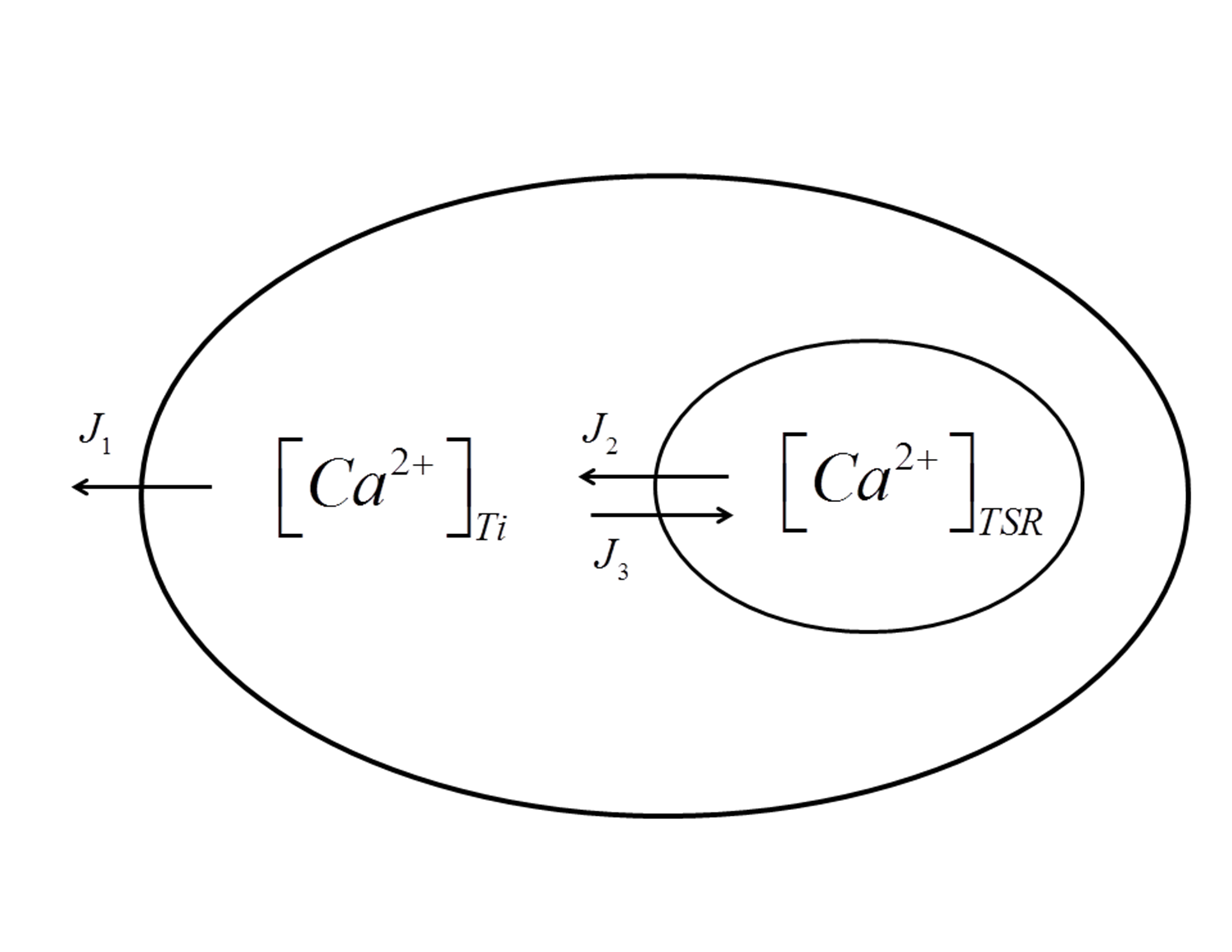

Supplement: S1 Fig — Graphical model of smooth muscle, not at scale, of the two intracellular compartments of interest, as well as those Ca2+ fluxes between these two compartments. (TIF) [file pone.0138195.s001.tif]

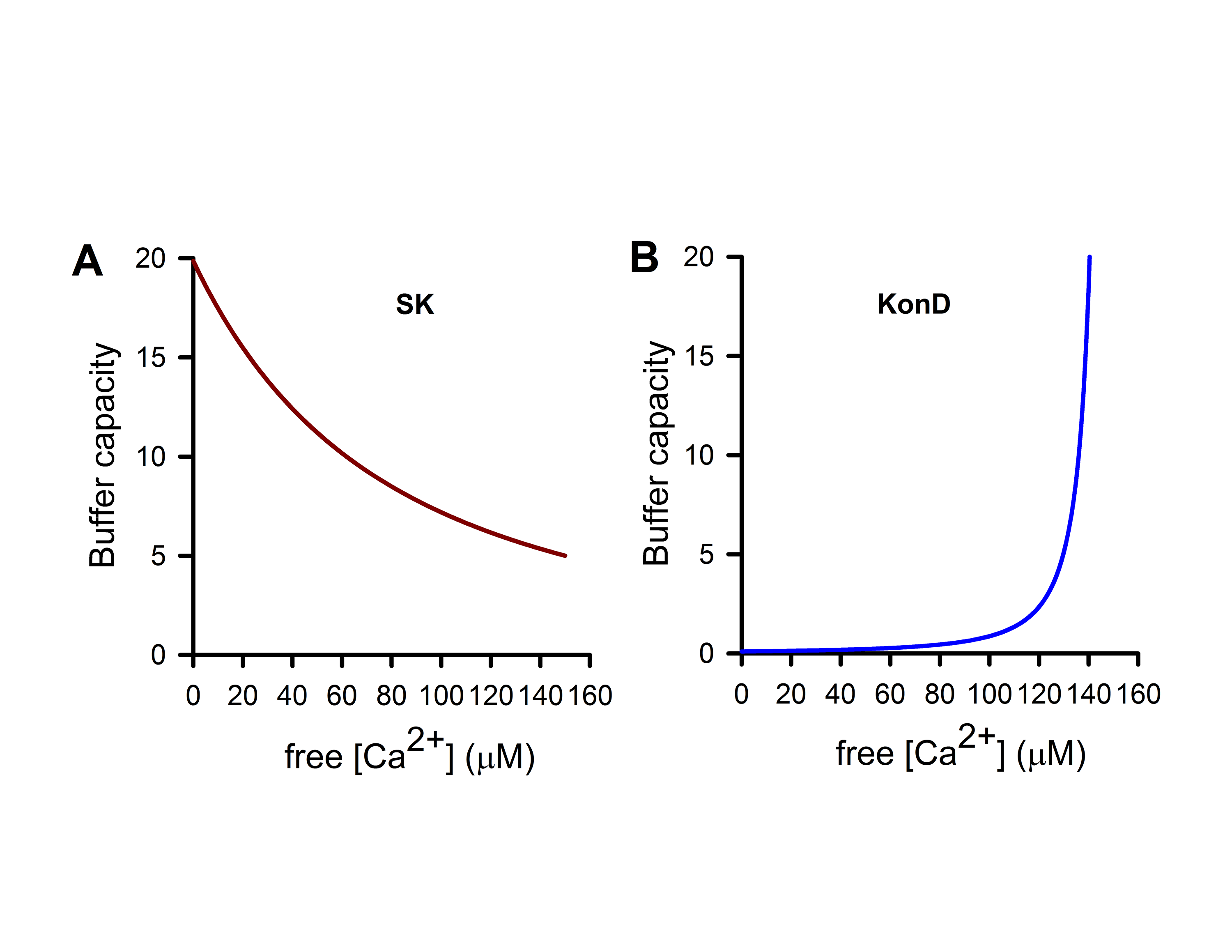

Supplement: S2 Fig — (A) Typical relationship for a 3 mM Ca2+ binding protein with a KD of 151.1μM using Eq (1.7). (B) Ca2+ buffering capacity for the same [Ca2+] as in A but using KonD model and Eq (1.19) with values indicated in the text. (TIF) [file pone.0138195.s002.tif]

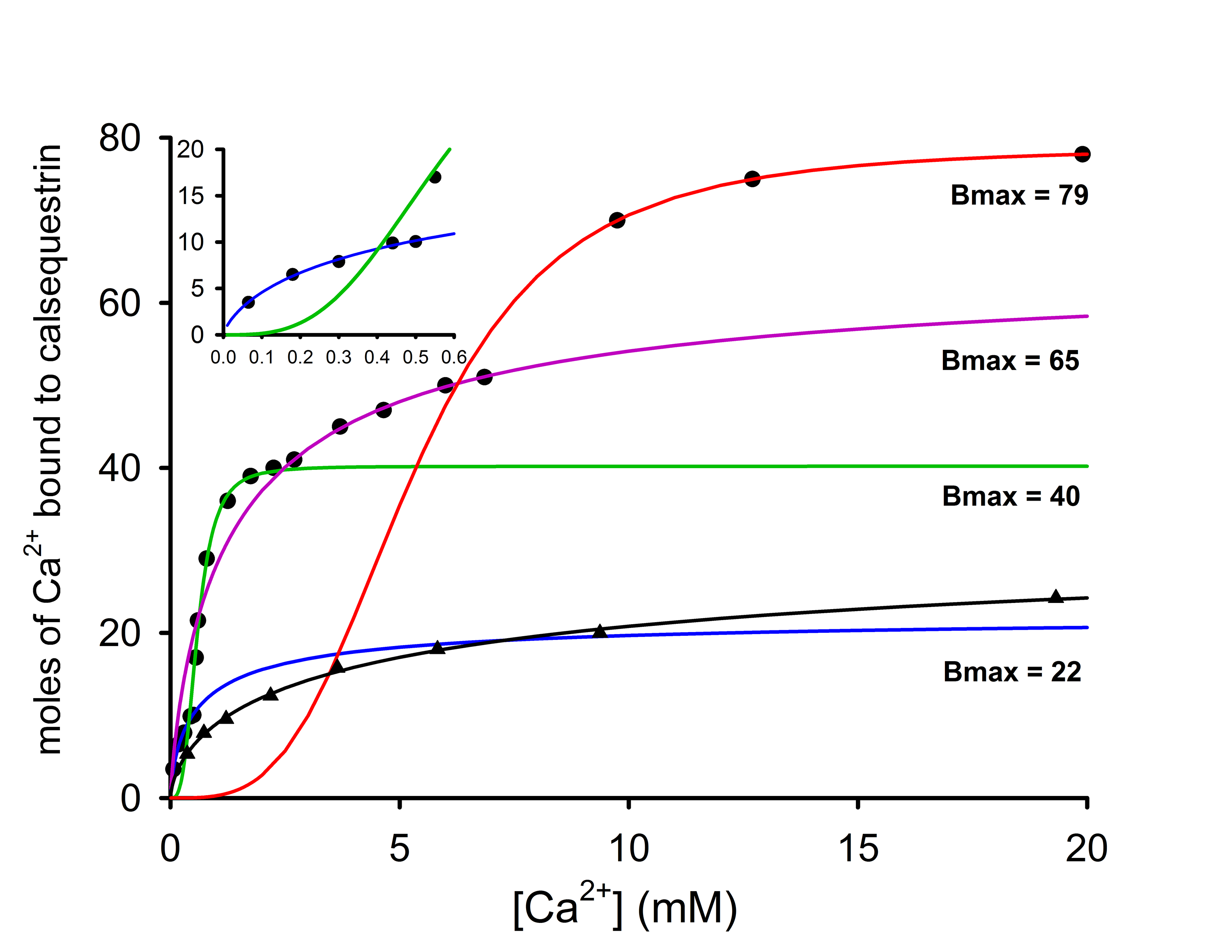

Supplement: S3 Fig — Models of Ca2+ bound to calsequestrin as a function of [Ca2+]; data replotted from Fig 5 of reference [28]. Data were fitted to a Hill equation. Fitting results suggests that the maximal number of Ca2+ binding sites (Bmax) is increasing as a function of the [Ca2+]. The inset shows the fitting for [Ca2+] in the range of 0–0.6mM. Park et al. have shown that blocking polymerization of calsequestrin inhibits this effect of increasing Bmax, so Bmax value stays around 22 for the whole range of [Ca2+] tested (triangle line) [28]. At least 5 points were used to calculate Bmax value higher than 70. (TIF) [file pone.0138195.s003.tif]

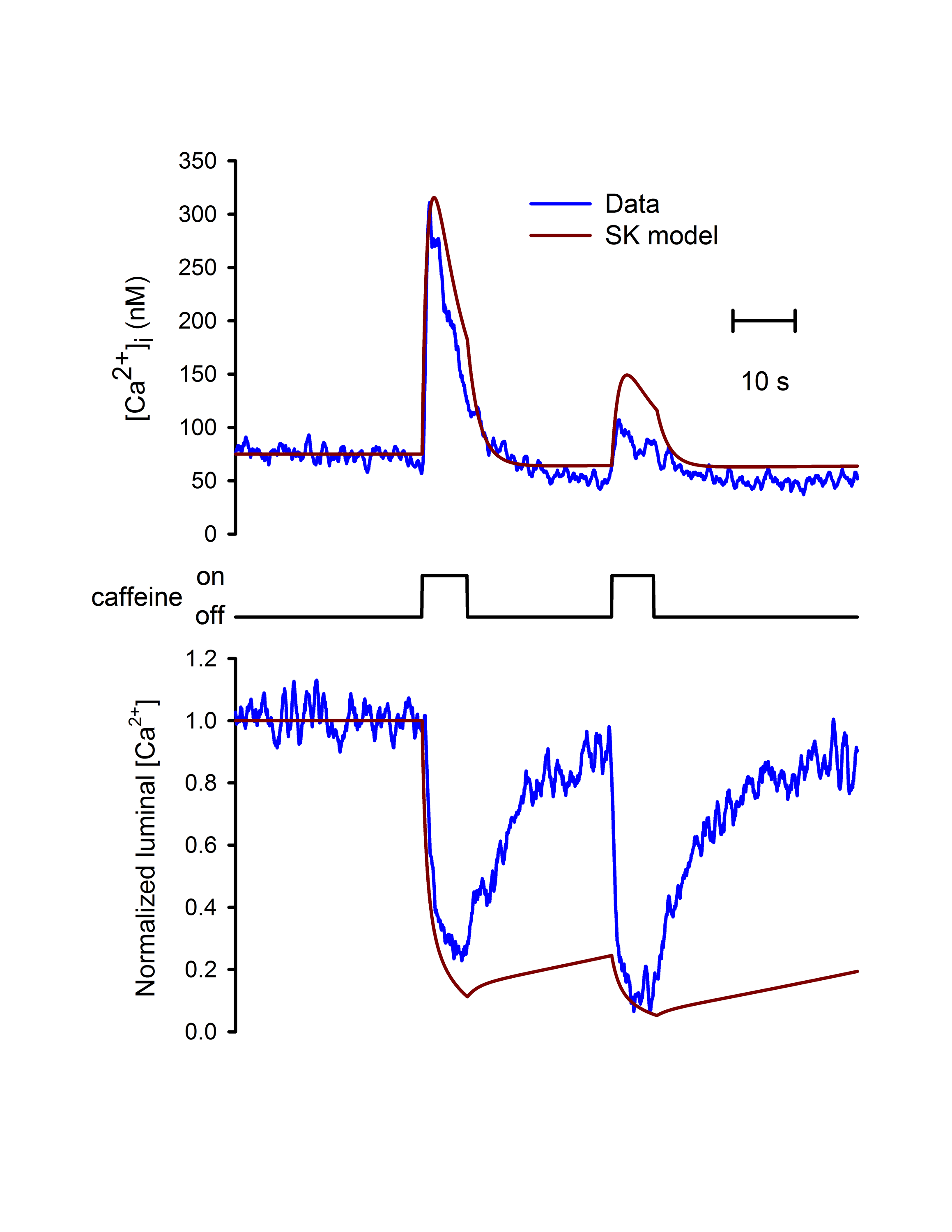

Supplement: S4 Fig — In this case the gamma value used in the SK model (red line) was modified to fit the caffeine-induced [Ca2+]i response (blue line). Although there is now a good fitting of the [Ca2+]i responses induced by caffeine, the time course of recovery of the [Ca2+]FSR is much slower in the SK model than is recorded by the Mag-Fluo-4 indicator. This can be explained by considering that free and total Ca2+ go together in the SK model, while in the KonD model (see Figs 5 and 7) there is a rapid recovery of the [Ca2+]FSR that precedes the recovery of the [Ca2+]TSR, which explains the presence of a refractory period due to the recovery of the [Ca2+]FSR but not of the total, so the second caffeine-induced Ca2+ response is much smaller. (TIF) [file pone.0138195.s004.tif]
