# Supplementary material for: Kinetics on Demand Is a Simple Mathematical Solution that Fits Recorded Caffeine-Induced Luminal SR Ca2+ Changes in Smooth Muscle Cells
Source: PLoS One. 2015 Sep 21;10(9):e0138195. doi: 10.1371/journal.pone.0138195 (PMC4577101; doi:10.1371/journal.pone.0138195)
Supplement: S1 Text — (DOCX) [file pone.0138195.s005.docx]

**Parameter estimation**

We have used experimental data, the 20 mM-caffeine induced [Ca^2+^]_i_ responses, which are reported in Fig 2 of this manuscript, to estimate some of the parameters that have been used to solve the present mathematical model. While the rest of the parameters were empirically estimated by searching for those values that allow the model to best reproduce the same experimental data (Fig 2). Below, you can find a detailed description on how these parameter values were estimated.

- $a.$ Being directly related to those pumps present at the plasma membrane that remove the excess of Ca^2+^ from the cytoplasm, this parameter largely determines the speed of the decay phase in the caffeine-induced Ca^2+^ response. The value that best agrees with our experimental results is

$$a=35 s^{-1}.$$

- $b.$ This parameter is very sensitive to the time at which caffeine-induced Ca^2+^ response has reached maximum value. The number selected was the one that best allowed to reproduce the rate of rise vs. rise time graph shown in Fig 2B is:

$$b=65 s^{-1}.$$

- $c.$ This parameter determines the rate of SR refilling with Ca^2+^ after caffeine-induced Ca^2+^ release based on data shown in Fig 2C. The final value was obtained by fitting data shown Fig 2:

$$c=11,250 nM/s.$$

- $\overline{\left[Ca^{2+}\right]}_{i}.$ This is the basal concentration of free cytoplasmic Ca^2+^ which has been experimentally measured in the range $75-100 nM$ [1]. Here we have taken

$$\left[Ca^{2+}\right]_{i}\approx75 nM.$$

- $\gamma.$ The ratio of volume between SR and cytoplasm has been found to take values in the following approximated range [2]:

$$\gamma\in\left[0.01,0.1\right].$$

- $n_{v}.$ The scaling coefficient that allows the model to best reproduce the cell-variability experiments (in particular the rate of rise vs. rise time graph shown in Fig 2B) is:

$$n_{v}\approx1.7.$$

- $K_{S}, n_{s}.$ The values for half saturation with Ca^2+^ and the Hill coefficient for SERCA pumps were taken from [3]:

$$K_{S}\approx300 nM, n_{s}\approx2.$$

- $\beta.$ This parameter represents the Ca^2+^buffering capacity of the cytoplasm and it has been shown that  *β* has a constant value in the physiological range of [Ca^2+^]_i_ responses in smooth muscle as reported in [4]. Although the *β* value experimentally determined showed large variability among different cells, we have arbitrarily taken this value:

$$\beta\approx100.$$

- $n_{F}, k_{f}.$ These parameters that shape the RyR open-probability function were empirically estimated so the model fits cell-variability experimental results (that is the rate or rise vs Δ[Ca^2+^]_i_ response graph shown in Fig 2A):

$$n_{F}\approx1.8, k_{f}\approx4 mM^{-1}.$$

- $K_{C}.$ This parameter, also involved in the definition of the open-probability function of RyR was computed in terms of other parameters in the model in order to comply with the idea that the resting values for both [Ca^2+^]_i_ and [Ca^2+^]_FSR_ should satisfy [5]:

$$\overline{\left[Ca^{2+}\right]}_{i}\approx75 nM, \overline{\left[Ca^{2+}\right]}_{FSR}\approx150 \mu M.$$

Since in the steady state $J_{2}=J_{3},$ this parameter is calculated by solving for K_C_ in such equality, after substituting $\left[Ca^{2+}\right]_{i}=\overline{\left[Ca^{2+}\right]}_{i},$ $\left[Ca^{2+}\right]_{FSR}=\overline{\left[Ca^{2+}\right]}_{FSR}$ and $\left[Caff\right]=0.$

- $K_{R}.$ This parameter represents the affinity of the luminal SR Ca^2+^ binding proteins and the selected value allowed us to reproduce both phase 1during Ca^2+^ release (Fig 6) and the fast recovery of the [Ca^2+^]_FSR_ (Fig 7):

$$K_{R}=151.5 \mu M.$$

- $P_{E}.$ Represents the number of Ca^2+^ binding sites in the absence of Ca^2+^. This parameter value was calculated assuming an SR luminal Ca^2+^ buffering capacity of approximately 10 [6]. In other words that $\left(\overline{\left[Ca^{2+}\right]}_{TSR}-\left[Ca^{2+}\right]_{FSR}\right)/\overline{\left[Ca^{2+}\right]}_{FSR}\approx10$. Thus, in the KonD situation we have that

$$P_{E}=10\left(K_{R}-\overline{\left[Ca^{2+}\right]}_{FSR}\right) \therefore P_{E}\approx15 \mu M,$$

while in the standard kinetics $P_{E}$ is calculated as

$$P_{E}=10\left(K_{R}+\overline{\left[Ca^{2+}\right]}_{FSR}\right) \therefore P_{E}\approx3 mM.$$

**REFERENCES**

1. Case RM, Eisner D, Gurney A, Jones O, Muallem S, Verkhratsky A. Evolution of calcium homeostasis: From birth of the first cell to an omnipresent signalling system. Cell Calcium. 2007;42: 345–350.

2. Gómez-Viquez NL, Guerrero-Serna G, Arvizu F, García U, Guerrero-Hernández A. Inhibition of SERCA pumps induces desynchronized RyR activation in overloaded internal Ca2+ stores in smooth muscle cells. Am J Physiol Cell Physiol. 2010;298: C1038–46.

3. Lytton J, Westlin M, Burk SE, Shull GE, MacLennan DH. Functional comparisons between isoforms of the sarcoplasmic or endoplasmic reticulum family of calcium pumps. J Biol Chem. 1992;267: 14483–14489.

4. Guerrero a, Singer JJ, Fay FS. Simultaneous measurement of Ca2+ release and influx into smooth muscle cells in response to caffeine. A novel approach for calculating the fraction of current carried by calcium. J Gen Physiol. 1994;104: 395–422.

5. Case RM, Eisner D, Gurney A, Jones O, Muallem S, Verkhratsky A. Evolution of calcium homeostasis: from birth of the first cell to an omnipresent signalling system. Cell Calcium. 2007;42: 345–50.

6. Dagnino-Acosta A, Guerrero-Hernández A. Variable luminal sarcoplasmic reticulum Ca(2+) buffer capacity in smooth muscle cells. Cell Calcium. 2009;46: 188–96.
